# Using Daily Diary Assessments to Better Understand the Role of Parental Consistency in the Development of Externalizing Child Behavior

**DOI:** 10.1007/s10802-023-01073-w

**Published:** 2023-05-19

**Authors:** Alithe Van den akker, Patty Leijten, Peter Hoffenaar, Frances Gardner

**Affiliations:** 1 University of Amsterdam, Research Institute of Child Development and Education, Amsterdam, The Netherlands; 2 Oxford University, Department of Social Policy and Intervention, Oxford, UK

**Keywords:** Consistency, Discipline, Disruptive behavior, Externalizing, Diary study

## Abstract

Consistent discipline is thought to reduce early child externalizing behavior. It is unclear, however, whether consistency is important mainly within episodes of misbehavior (e.g., threatening with discipline but then giving in) or across episodes of misbehavior (e.g., disciplining each instance of misbehavior). Using a daily diary approach, we examine whether these two types of consistency are associated with disruptive child behavior, concurrently and prospectively. We included two samples (Sample 1: *N* = 134, *M*_agechild_ = 30 months, 44% girls; Sample 2: *N* = 149, *M*_agechild_ = 5.88 years; 46% girls, at-risk sample) with daily reports of child disruptive behavior and parental responses (Sample 1 = 7 days; Sample 2 = 14 days). Sample 1 parents additionally reported on their reactions over the past month and their child’s externalizing behavior one year later. Within-episode consistency was assessed by the average number of parental reactions per episode; across-episode consistency by the Index of Qualitative Variation; and general consistency by parents’ report of how they had responded to child disruptive behavior in the past month. In both samples correlations between within- and across-episode consistency were significant, but not so strong that they were not differentiated. Again in both samples, regression analyses provided evidence for unique predictive value of across-episode, not within-episode, consistency for daily disruptive behavior. Parental general consistency was longitudinally associated with fewer externalizing problems, whereas within- and across-episode consistency were not. It appears meaningful to differentiate within- from across-episode consistency to better understand the relevance of different aspects of consistency.

## Introduction

Many studies support the notion that when parents are consistent in their responses to their child’s misbehavior, children show less externalizing behavior (Barry et al., 2009; Gardner, 1989; Gryczkowski et al., 2010; Halgunseth et al., 2013; Lengua & Kovacs, 2005). However, these studies have focused on global self-reports, which mostly confound consistency of parental reactions across multiple episodes of misbehavior (e.g., punishing misbehavior in one episode, and condoning it in another) with consistency within a single episode of misbehavior (e.g., threatening with punishment, but leaving it in the end). Additionally, observational studies have mostly focused on within-episode consistency (Del Vecchio & O’Leary, 2006; Gardner, 1989). Therefore, it is not known whether within- and across-episode consistency play similar roles in the early development of externalizing problems. In this study, we use a daily diary approach to examine parental consistency both within and across episodes, offering a unique possibility to differentiate these two types of consistency. We examine associations between these aspects of consistency, and how they are associated with child externalizing behavior, both concurrently and longitudinally.

### Differentiating Within- from Across Disruptive Behavior Episode Consistency

Empirically, studies using questionnaire measures of inconsistent discipline have indeed found it to be associated with more externalizing behavior in children (Barry et al., 2009; Gryczkowski et al., 2010; Halgunseth et al., 2013; Lengua & Kovacs, 2005). However, research to date has not separated within-episode consistency from across-episode consistency. For instance, the frequently used Alabama Parenting Questionnaire (Frick et al., 1999) includes items in the inconsistency scale asking parents whether they threaten to punish, but then do not do so in the end, or whether they let their child out of punishment early, which are examples of inconsistent responding *within* a single episode of misbehavior. Additionally, items are included asking parents whether their punishment depends on their mood, which concerns consistency *across* episodes of misbehavior. It is important to note that these types of inconsistency do not necessarily co-occur. Parents may be relatively inconsistent in their responses within a single episode, perhaps not feeling competent enough to follow through with an initial course of action and giving up along the way (Deković et al., 2010), yet they may be consistent in this response style across multiple episodes of misbehavior. Alternatively, parents may be consistent within an episode of misbehavior by providing a negative consequence and sticking to it, but respond in different ways to new episodes, for instance switching to ignoring the misbehavior or trying to redirect attention by making a joke. They may switch approaches because they are in a different mood state themselves (Rueger et al., 2011), or because they felt their approach was not effective in a previous instance.

Several theoretical frameworks would predict that both types of consistency would be associated with increased externalizing problems. Attachment theory postulates that children form secure attachments to their caregivers if these are consistently responsive to their needs. With consistency, children learn that they can trust their caregiver to provide them with a secure base and a safe haven (Ainsworth et al., 2015). With inconsistency in caregiving, in contrast, children learn that their environment is unpredictable and insecure, increasing the risk of attachment problems and problem behavior (Madigan et al., 2016). Supporting this notion, unpredictable behavior from parents has been shown to impact the stress response in both very young children (Noroña-Zhou et al., 2020), as well as older children (Manczak et al., 2018). A dysregulated stress system may in turn result in problems with self-regulation, resulting in heightened disruptive behavior (Wesarg et al., 2020). Additionally, unpredictability likely hampers children’s ability to develop a sense of self-efficacy, because it prevents them from developing a sense of control over situations (Bandura, 1978; Lippold et al., 2016). Low self-efficacy, in turn, makes it more difficult to regulate anger and frustration, resulting in increased levels of disruptive behavior.

Social learning theories emphasize operant conditioning principles which predict that within-episode inconsistency would be associated with increased problematic behavior because failing to follow-through with negative consequences reinforces the child for showing the misbehavior (Patterson, 1982). Additionally, consistent negative consequences across different disruptive behavior episodes, or at least a lack of reward, would quickly result in extinction of problematic behavior, whereas inconsistent discipline – with intermittent patterns of positive or negative reinforcement – makes it difficult for children to learn that their behavior is not acceptable. Both social learning and attachment frameworks would thus predict that within- as well as across-episode consistency would play a role in the maintenance of disruptive behavior.

Although most questionnaire studies have confounded within- and across-episode consistency, some studies have specifically investigated within-episode consistency. Using observations of parent-child interactions, parents with children who were high on externalizing behavior were less likely to follow-through on an initial demand than parents of children who were low on externalizing behavior (Gardner, 1989). Additionally, mothers of aggressive toddlers have been observed to be more likely to react with both overreactivity and laxness to instances of child aggression than mothers of non-aggressive toddlers (Del Vecchio & O’Leary, 2006). However, with observations it is more difficult to assess how this within-episode consistency relates to across episode consistency. As observations are already so time-consuming, observing (enough) episodes of misbehavior to examine across episode consistency would be especially difficult.

### Using Daily Diaries to Assess Parental Consistency

Daily diary assessments allow for overcoming the abovementioned limitations of single-time surveys or observations. Single-time self-reports of parental consistency may be biased, as correctly judging how consistent one is in their reactions might be even more difficult than broadly gauging whether one frequently reacts in a certain way (Lippold et al., 2016). A more valid way to assess parental consistency may therefore be to repeatedly ask parents about their actual behavior, and analyze the consistency across their responses. A small scale study examined parents’ daily fluctuations in their overreactive and lax discipline, with lax discipline indicating inconsistency by not following through. Although overreactivity and laxness were positively correlated at the between-person level, indicating that mothers who were generally more overreactive were also more lax, there was a negative association at the within-person level, indicating that when mothers were overreactive in a certain instance, they were less likely to be lax at that time, and vice versa (Passini et al., 2013). These results indicate that mothers can be inconsistent across episodes with regards to their within-episode consistency. However, associations with child externalizing behavior were not examined here. Another diary study did examine associations with externalizing behavior and found that for mothers with 5- 8-year old children maternal consistency across one week was associated with less child externalizing behavior (Villarreal et al., 2021). Consistency was operationalized as the within-person fluctuations in destructive conflict characteristics, which included both maternal punitive behavior as well as child and mother negativity, making it unclear whether this was an association with inconsistency in maternal behavior specifically. Yet another study examined daily reports of parents harsh and warm reactions to child misbehavior and found that consistency in the level of warmth was associated with less child ADHD symptoms, whereas consistency in the level of harshness was not (Li & Lansford, 2018). As these studies operationalized inconsistency as fluctuations in mean levels of parenting behaviors, we do not know whether parents exhibited different types of responses in the same instance of misbehavior. For instance, some parents may be more likely to only punish the child by taking away a privilege, whereas other parents are more likely to yell at the child, take away a privilege and also comfort the child, with the latter type of response perhaps especially confusing to the child. A previous observational study has indeed found that some parents were more likely to use both positive and negative discipline strategies within the same episode than others, but did not examine associations between this inconsistency and externalizing behavior (van Zeijl et al., 2007).

### The Present Study

In the present study, we use daily diary data to differentiate consistent responding within a single episode of misbehavior from consistent responding across multiple episodes of misbehavior and examine how they are each associated with the severity of disruptive behavior in children. We assess within-episode consistency as the mean number of different reactions to a specific episode of misbehavior, distinguishing positive attention, positive consequence, negative consequences, negative attention, and ignoring. Across-episode consistency is assessed as the overall dispersion of mothers’ reactions across all possible categories, taking into account the total number of episodes of misbehavior across a week. Parental reactions that were concentrated in fewer reaction categories were indicative of more consistency.

We examine associations between the two types of consistency in two independent samples: a community sample (*N* = 134) of mothers of 1.5 to 3.5 year old children who completed a daily diary for 7 days, and an at-risk sample with heightened disruptive behavior (*N* = 149) of 3 to 8 year old children who filled out a daily diary for 14 days. Including these two samples has several benefits. First, we are able to examine whether associations between the two types of consistency conceptually replicate across multiple samples. Second, we can investigate whether both types of consistency are equally associated with disruptive behavior during a developmental stage when disruptive behavior starts to emerge and is relatively more normative, as during a developmental stage when disruptive behavior for most children has started to decline (Tremblay, 2010) as a result of increases in children’s verbal-skills and overall self-regulation (Kuhn et al., 2016). This allows us to investigate whether the two types of consistency play a similar role in the early emergence as in the maintenance of more persistent problem behavior across development. Contemporary accounts of social learning theories would predict that across-episode consistency may be less relevant for problem behavior that persists into preschool age, as repeated coercive cycling in parent-child dyads is thought to result in increasingly rigid, mutually negative interactions over time (Granic & Patterson, 2006).

We also examine the added value of computing these types of consistency from daily diary data over a single questionnaire assessment. To this end, we assessed whether it is a better predictor of children’s externalizing behavior one year later than a measure of consistency derived from a general questionnaire as administered in a single – less time-consuming baseline assessment – asking parents to estimate how often in the past month they showed the reactions that we also included in the diary study. Although this ‘general consistency’ measure confounds within- and across-episode consistency, it may still be a better measure of consistency than some of the current measures that are used. Rather than asking parents to report on how consistent they are, we merely asked parents to indicate how often they reacted a certain way, and compute consistency by calculating the dispersion of parents’ responses across the different reaction categories. This approach makes it less likely that this association is for instance explained by parents scoring themselves as inconsistent due to a more negative self-view (Smit et al., 2021). This likely plays a role in more traditional questionnaire measures, as most parents will realize that threatening with punishment and then not following through is not an effective parenting strategy. Our approach will allow us to examine whether taking multiple days of measurements to assess consistency is really necessary, or whether we have enough information when we just ask parents how often they react a certain way overall. Additionally, associations between the general consistency and within- and across-episode consistency can be examined to provide an indication of the validity of these measures.

## Method

### Sample

We included two samples, to allow for conceptual replication: Sample 1 is a community sample of 134 mothers of 1.5–3.5 year old children (*M* = 30 months, 44.3% girls), who reported on their child’s temper tantrums (frequency and severity) and their responses to these tantrums – in general across the past month and daily for 7 days. Mothers were predominantly, but not exclusively, highly educated (79% higher vocational or university education), and were not selected for experiencing any particular difficulties with their child. Seven percent indicated that they raised their child without a partner. No information regarding ethnicity was collected for this sample.

Between February 2016 and June 2017, undergraduate students recruited mothers with children between one and five years old for a research practical. They recruited mothers through online parenting fora and Facebook, and face-to-face outside in Amsterdam. Mothers who participated were also asked to forward the invitation for the study to other mothers. Mothers were informed about the study and gave informed consent in the online study environment. They filled out the general questionnaire regarding: children’s temper tantrums and their own reactions, their personality and sense of parenting competence (*N* = 884). Mothers who indicated that their child was between 1.5 and 3.5 years old were asked if they would like to participate in an additional daily diary study, and *N* = 382 indicated that they would like to receive more information. They were contacted by telephone, with *N* = 220 eventually participating. For this study, we only selected participants if they had participated in at least 4 days of the study (*N* = 185), and who had reported reactions for at least two tantrums, resulting in a final sample of *N* = 134. Participants completed an average of 6.76 days (*SD* = 0.62, range = 4–7 days). For this sample, mothers additionally reported on their child’s externalizing behavior one year later (*n* = 86). Participants who dropped out of the study did not differ significantly from those who participated one year later with regards to age of the mother (*T*(131) = 1.66, *p* = 0.062) or child (*T*(132) = -1.65, *p* = 0.050), the child’s sex (*χ*2(1) = 0.29, *p* = 0.589) or mothers’ educational level (*χ*2(4) = 0.74, *p* = 0.947). Additionally, there were no significant differences in children’s tantrum severity (*T*(132) = 0.90, *p* = 185) and mothers’ within-, across-, or general consistency at T1 (*T*(132) = -0.10,* p* = 0.922; *T*(132) = -0.09, *p* = 0.930; *T*(117) = -0.21, *p* = 0.833, respectively).

Mothers who filled out the general questionnaire had a chance of winning a gift certificate of 50 euros. Mothers who participated at least four days of the diary study received a small gift (a small book for their child – 2 euros) by mail. The study was approved by the ethical review board of the Department of Child Development and Education at the University of Amsterdam (#2015-CDE-6367).

Sample 2 consists of 149 parents (94% mothers) of 3–8 year old children (*M* = 5.88; 46% girls) oversampled for disruptive behavior – 17% had received parenting support for disruptive child behavior prior to the study; seven percent still received support during the study. Parents reported on how they generally responded to their child’s disruptive behavior as well as their daily responses for 14 days. Parents were predominantly, but not exclusively, highly educated (78% higher vocational or university education). Ten percent indicated that they raised their child without a partner. Culturally, 93% identified as Dutch, of which 19% identified as bicultural (mainly other European cultures or Moroccan). Others self-identified as Moroccan, other European, Asian, Surinamese, or Turkish. This roughly represents the Dutch population where around 25% of families has at least parts of their roots outside the Netherlands, most often in Turkey, Morocco and Surinam (Centraal Bureau voor de Statistiek [CBS], 2020).

Parents were recruited between March 2020 and June 2021, through social media, primary schools across the Netherlands, and databases from the University of Amsterdam of parents who consented to be contacted for research projects. Children with disruptive behavior problems were oversampled by advertising the study as targeting parents of children with mild to moderate levels of disruptive behavior. Parents who signed up were contacted by phone to explain the study procedures. Parents who agreed to participate signed informed consent, completed a baseline assessment (i.e., demographics and trait measures) with a link to daily online daily questionnaire (*N* = 156). For this study, we only selected participants if they had participated for at least 8 days of the study, and who had reported reactions to at least two disruptive behaviors, resulting in a final sample of *N* = 149. Participants completed an average of 13.22 days (*SD* = 1.34, range = 8–14 days).

Parents received €50 for completing the study. Study procedures were approved by the Ethical Review Board of the department of Child Development and Education of the University of Amsterdam (2019-CDE-11055).

### Measures

#### Parental Consistency

In Sample 1, parents reported on how they responded to their child’s tantrums both in general over the past month – before they started the diary study, and for each tantrum that took place during the diary study (for a maximum of seven tantrums a day). Parents rated their responses from a list of 11 behaviors. We made a functional classification based on social learning principles (Patterson, 1982), differentiating punishment and reward from lack of punishment or reward, and positive and negative attention: negative consequence (2 items: ‘I sent my child to their room/corner/time-out’, ‘I punished my child’), positive consequence (‘I negotiated with my child’, ‘I gave in to my child’), withholding attention (‘I didn’t, I let my child cool off’, ‘I ignored my child’), negative attention (‘I became angry with my child’, ‘I grabbed my child’, ‘I spoke sternly to my child’), positive attention (‘I distracted my child’, ‘I comforted my child’). In the questionnaire about responses to tantrums in general, participants indicated how often they tended to respond that way (1 = *never*; 2 = *almost never*; 3 = < *half the time*; 4 = *about half the time*; 5 = > *half the time*; 6 = *almost always*; 7 = *always*), and we computed a mean score per category. In the daily diaries parents indicated whether or not they responded that way in that particular instance (0 = *no*; 1 = *yes*), allowing for multiple responses. Parents received a score of 1 in a category when answered yes to at least one of the responses in that category.

For our measure of across-episode consistency from the daily diary data and the general consistency measure from the baseline questionnaire, we calculated the Index of Qualitative Variation (IQV), which is a measure of variation for nominal variables – where a standard deviation cannot be computed due to qualitative rather than quantitative differences between categories, using the following formula (Frankfort-Nachmias & Leon-Guerrero, 2018):

$$\mathrm{IQV}=\frac{\left(\mathrm{K}\left(1-\left(\sum \mathrm{Prop}2\right)\right)\right)}{\left(\mathrm{K}-1\right)}$$

For each response category we first computed what proportions of the total number of responses they were for each individual. The squared proportions of each of the categories are summed and then subtracted from 1 and multiplied by K, which is the number of categories (5 in our study). This is then divided by the number of categories minus 1. The resulting value can range from 0 to 1.00, with higher scores indicating greater inconsistency. Therefore, we subtracted this value from 1, so that higher scores indicated greater consistency.

From the diary data, we additionally computed a measure of within-episode consistency. For each tantrum, we summed the total number of responses in the different categories (potential range 1–5), and then computed a mean score across all tantrums that were reported during the study. The observed range was 1–3 with higher values indicating less consistency. For ease of interpretation we recoded this variable so that higher values indicated greater consistency by subtracting the values from 3. The final variable thus ranged from 0–2. The intraclass correlation coefficient (ICC) for within-episode consistency was 0.20. This value is similar to ICCs that have previously been reported for parenting variables in diary studies, such as psychological control and autonomy support (Mabbe et al., 2018), with somewhat higher levels of around 0.33 also reported for psychological control (Aunola et al., 2013).

In Sample 2, parents were asked how they responded to their child’s disruptive behavior in general at the start of the study, and how they responded to their child’s most challenging disruptive behavior that particular day (if any) in the diary study. Parents rated their responses from a list of 13 behaviors, which we grouped into the same five categories as for Sample 1: negative consequence (4 items: ‘I sent my child to their room for at least an hour’, ‘I gave my child a short time-out, away from others’, ‘I took something nice away from my child (e.g., toys or screen time)’, I gave my child extra chores (e.g., set the table)’), positive consequence (2 items: ‘I gave my child his/her way’, ‘I gave in to my child’), withholding attention (2 items: ‘I did nothing’, ‘I talked about it with my child afterwards’), negative attention (3 items: ‘I yelled/swore’, ‘I said things I didn’t mean’, ‘I threatened with punishment, but did not punish’), positive attention (2 items: ‘I begged my child to stop’, ‘I used humor to distract my child’). In the general questionnaire, participants indicated how often they tended to respond that way on a 5-point Likert type scale (1 = *less than once a week,* 2 = *once a week*; 3 = *few times a week*; 4 = *once a day*; 5 = *several times a day*), and we computed a mean score per category. In the daily diaries study parents indicated whether or not they responded that way in that particular instance (0 = *no*, 1 = *yes*), allowing for multiple responses. Parents received a score of 1 in a category when answered yes to at least one of the responses in that category. When parents reported that their child had not shown any disruptive behavior that day, the response category was coded as missing.

Like in Sample 1, we computed the IQV as a measure of across-episode consistency from the daily diary data and a measure of general consistency from the baseline questionnaire, and the mean number of selected categories of responses per episode as our measure of within-episode consistency. For within-episode consistency, the ICC was 0.18.

#### Child Externalizing Behavior

In Sample 1, we calculated a measure of severity of the child’s tantrum behavior from the daily diary reports, by summing for each tantrum the total number of aggressive (hitting, kicking, biting, throwing an object, pushing/pulling, spitting, grabbing) and self-injurious behaviors (banging head, holding breath, freezing). A previous study on this sample found that a profile with elevated levels on these behaviors was predictive of both internalizing and externalizing problems above and beyond tantrum frequency and duration (Van den Akker et al., 2022). The ICC for tantrum severity was 0.24.

One year later (T2), parents in Sample 1 filled out 24 items of the Externalizing Problem Behavior Scale (the attention problem and aggressive behavior problem subscales, e.g., “My child does not seem to feel guilty after misbehavior”) of the Dutch version of the Child Behavior Checklist (1, 5–5) (Achenbach & Rescorla, 2000). Parents were instructed to indicate for the past 2 months how characteristic the item was of their child's behavior, with each item rated as 0 (*not true*), 1 (*sometimes/somewhat true*), or 2 (*often/very true*). Cronbach's alpha for the present sample was 0.89.

For Sample 2, rather than indicating how many disruptive behaviors children had displayed, parents rated children’s overall level of disruptive child behavior at T1 each day (i.e., “how disruptive was your child’s behavior today?”) on a 1 − 10 scale. A mean score across the 14 days was computed. The ICC was 0.32.

### Analysis Plan

Hypotheses and analyses were registered on the Open science Framework (https://osf.io/tecr4/?view_only=0c6f1e3d6b2f46e49c5599c4c168be3c).

We first winsorized outliers (outside 1.5* IQR) to the nearest value if there was a gap in data between that range and the outlier. In Sample 1, for the within-episode consistency measures as derived from the daily diaries we identified two outliers, and for the across-episode consistency derived from the questionnaire asking about tantrums in general, we identified one outlier. For the severity of daily disruptive behavior we identified six outliers, and for externalizing behavior we identified two outliers. In Sample 2, for the within-episode consistency measure as derived from the daily diaries we identified one outlier, and for the across-episode consistency we identified four outlier. For the severity of daily disruptive behavior we identified four outliers. To answer our first research question- whether our measures of within- and across-episode consistency measure different but related aspects of consistency – we computed correlations. Next, we performed regression analyses to predict the severity of daily disruptive behavior from the within- and across-episode consistency measures to see whether they were uniquely associated. In a next step, we examined whether associations were significant above and beyond mean levels of the daily parental reactions. These analyses control for child sex and age and parental educational level and are performed on both Samples 1 and 2. As 11 parents in Sample 2 received parenting support for their child’s behavior, we also controlled for received support in Sample 2. Finally, we performed regression analysis in SPSS (version 28) to examine – in Sample 1 – whether within- and across- episode consistency as derived from the daily diary reports longitudinally predict child externalizing behavior problems one year later (T2), over and above a measure of consistency derived from estimates of parental behavior across the past month, controlling for the severity of temper tantrum behavior as reported in the diary study at T1.

## Results

On average, children in Sample 1 had an average 5.90 tantrums during the 7-day period (*SD* = 4.21, range 2–20), and for children in Sample 2 the mean level of disruptive behavior was rated 3.32 on the 10 point scale across the 14 days (*SD* = 1.21, range 1.14–6.54). Descriptives and intercorrelations for Samples 1 and 2 are provided in Table 1. In both samples, within- and across episode consistently were significantly associated. Associations were strong, but not so strong as to indicate that they would actually be measuring the same thing. In Sample 1, only across-episode consistency was negatively associated with disruptive behavior severity; in Sample 2, both within- and across-episode consistency were negatively associated with disruptive behavior severity.

**Table 1:** Descriptives and Intercorrelations for the Study Variables

Measures	1.	2.	3.	4.	5.	6.	7	8.	9.	10.	*M(SD)*
1.Within-episode consistency	–	0.53**	–	-0.28**	–	-0.34**	-0.31**	-0.27**	-0.50**	-0.56**	1.81(0.35)
2.Across-episode consistency	0.60**	–	–	-0.31**	–	-0.40**	-0.42**	0.20*	-0.44**	-0.38**	0.25(0.18)
3.General consistency	0.40**	0.49**	–	–	–	–	–	–	–	–	–
4.Daily disruptive behavior severity	-0.11	-0.29**	-0.16	–	–	0.30**	0.16	0.03	0.30**	0.19**	3.32(1.21)
5.Externalizing problems T2	-0.08	-0.22*	-0.32**	0.34**	–	–	–	–	–	–	–
6.Negative consequence	-0.23**	-0.24**	-0.31**	-0.03	0.10	–	0.25**	-0.06	0.11	-0.04	0.27(0.25)
7.Positive consequence	-0.34**	-0.34**	-0.21*	-0.03	-0.03	0.05	–	-0.05	0.15	0.12	0.08(0.11)
8.Withhold attention	-0.20*	-0.30**	-0.16	0.12	0.04	-0.09	-0.24**	–	-0.05	0.12	0.51(0.24)
9.Negative attention	-0.43**	-0.29**	-0.33**	0.03	0.20	0.22*	-0.00	0.01	–	0.17*	0.18(0.17)
10.Positive attention	-0.22*	0.09	0.30**	-0.09	-0.08	-0.17	0.00	-0.17	-0.18*	–	0.20(0.22)
*M(SD)*	1.52	0.40	0.07	4.15	0.60	0.07	0.12	0.30	0.21	0.42	–
	(0.47)	(0.29)	(0.05)	(4.04)	(0.31)	(0.16)	(0.21)	(0.28)	(0.26)	(0.30)	

Estimates for Sample 1 are provided below the diagonal, and for Sample 2 above the diagonal

*p < 0.05, **p < 0.01

### Within- and Across-episode Consistency and Severity of Child Disruptive Behavior

To examine whether within- and across-episode consistency were uniquely associated with the severity of daily disruptive behavior, we performed regression analyses. In Sample 1, the first step, controlling for age and sex of the child and educational level of the parent was not significant (*F*(3,130) = 0.47, *p* = 0.707, *R*^*2*^ = 0.01). Adding across-episode and within-episode consistency resulted in a significant improvement of the model (Δ*F*(2,128) = 6.95, *p* = 0.001, Δ*R*^*2*^ = 0.10): when parents were more consistent across disruptive behavior episodes, children displayed less severe disruptive behavior, whereas within-episode consistency was not significantly associated with severity of daily disruptive behavior (Table 2).

**Table 2:** Results of Regression Analyses Predicting Severity of Daily Disruptive Behavior in Sample 1

				Severity of daily disruptive behavior			
variables	*b(SE)*	β	*p*	variables	*b(SE)*	β	*p*
Step 1				Step 1			
age child	-0.04(0.04)	-0.08	0.370	Age child	-0.04(0.04)	-0.08	0.370
Sex child	-0.41(0.72)	-0.05	0.572	Sex child	-0.41(0.72)	-0.05	0.572
Education Level	-0.23(0.38)	-0.05	0.553	Education Level	-0.23(0.38)	-0.05	0.553
Step 2				Step 2			
Age child	-0.04(0.04)	-0.12	0.294	Age child	-0.04(0.04)	-0.08	0.385
Sex child	-0.65(0.69)	-0.07	0.353	Sex child	-0.30(0.73)	-0.04	0.685
Education Level	-0.24(0.37)	-0.05	0.525	Education Level	-0.14(0.40)	-0.03	0.725
Within-episode consistency	0.70(0.94)	0.06	0.455	negative consequence	-0.77(2.40)	-0.03	0.748
Across-episode consistency	-4.92(1.47)	-0.36	0.001	positive consequence	0.30(1.79)	0.02	0.866
				withholding attention	1.39(1.33)	0.10	0.297
				negative attention	0.56(1.47)	0.04	0.705
				positive attention	-0.96(1.27)	-0.07	0.454
				Step 3			
				Age child	-0.02(0.04)	-0.04	0.644
				Sex child	-0.80(0.71)	-0.10	0.259
				Education Level	-0.33(0.38)	-0.08	0.382
				negative consequence	-4.00(2.42)	-0.16	0.101
				positive consequence	-4.05(2.06)	-0.21	0.051
				withholding attention	-1.74(1.51)	-0.12	0.252
				negative attention	-1.78(1.65)	-0.11	0.282
				positive attention	-1.50(1.38)	-0.11	0.282
				Within-episode consistency	-0.55(1.17)	-0.06	0.638
				Across-episode consistency	-6.05(1.67)	-0.44	< 0.001

Results of Sample 2 conceptually replicated the findings of Sample 1. The first step, controlling for sex of the child and educational level of the parent was not significant (*F*(3,144) = 0.32, *p* = 0.808, *R*^*2*^ = 0.01). Adding across-episode and within-episode consistency resulted in a significant improvement of the model (Δ*F*(2,142) = 9.66, *p* < 0.001, Δ*R*^*2*^ = 0.12): only across-episode consistency, not within-episode consistency, was significantly associated with severity of daily disruptive behavior (Table 3).

**Table 3:** Results of Regression Analyses Predicting Severity of Daily Disruptive Behavior in Sample 2

				Severity of daily disruptive behavior			
variables	*b(SE)*	β	*p*	variables	*b(SE)*	β	*p*
Step 1				Step 1			
Age child	-0.00(0.01)	-0.03	0.736	Age child	-0.00(0.01)	-0.01	0.736
Sex child	-0.12(0.20)	-0.05	0.541	Sex child	-0.12(0.20)	-0.05	0.541
Education Level	0.06(0.10)	0.05	0.562	Education Level	0.06(0.10)	0.05	0.562
Received support	0.71(0.38)	0.16	0.062	Received support	0.71(0.38)	0.16	0.062
Step 2				Step 2			
Age child	-0.00(0.01)	-0.01	0.988	Age child	0.00(0.01)	0.02	0.776
Sex child	-0.09(0.19)	-0.04	0.639	Sex child	-0.06(0.19)	-0.02	0.770
Education Level	0.03(0.10)	0.02	0.785	Education Level	0.05(0.10)	0.04	0.612
Received support	0.83(0.36)	0.18	0.021	Received support	0.98(0.36)	0.21	0.007
Within-episode consistency	-0.59(0.32)	-0.17	0.063	negative consequence	1.45(0.39)	0.30	< 0.001
Across-episode consistency	-1.60(0.61)	-0.24	0.010	positive consequence	0.07(0.90)	0.01	0.938
				withholding attention	0.04(0.39)	0.01	0.901
				negative attention	1.77(0.54)	0.26	0.001
				positive attention	0.83(0.44)	0.15	0.060
				Step 3			
				Age child	0.00(0.01)	0.02	0.785
				sex	-0.05(0.19)	-0.02	0.778
				Education Level	0.04(0.10)	0.03	0.686
				Received support	0.96(0.36)	0.21	0.008
				negative consequence	1.42(0.46)	0.29	0.002
				positive consequence	-0.06(0.97)	-0.01	0.955
				withholding attention	0.22(0.44)	0.04	0.624
				negative attention	1.77(0.67)	0.26	0.009
				positive attention	0.86(0.59)	0.16	0.142
				Within-episode consistency	0.26(0.46)	0.08	0.578
				Across-episode consistency	-0.60(0.74)	-0.09	0.421

In a next set of regression analyses, we examined whether within- and across-episode consistency predicted the severity of daily disruptive behavior, above and beyond mean levels of the different response categories. In Sample 1, adding the mean levels of the proportions of the five parental responses across the seven days did not result in a significant improvement over the model including only age and sex of the child and educational level of the parent (Δ*F*(5,125) = 0.47, *p* = 0.801, Δ*R*^*2*^ = 0.02), indicating that how much parents displayed a certain type of reaction was not predictive of the child’s disruptive behavior. Adding within- and across-episode consistency to the model did result in a significant improvement (Δ*F*(2,123) = 8.63, *p* < 0.001, Δ*R*^*2*^ = 0.12). Across-episode consistency was predictive of daily disruptive behavior severity, whereas within-episode consistency was not. For model coefficients, see Table 2.

Different from Sample 1, in Sample 2, adding the mean levels of the proportions of the five parental responses did result in a significant improvement over the model including only sex and educational level of the parent (Δ*F*(5,139) = 6.21, *p* < 0.001, Δ*R*^*2*^ = 0.18). Providing negative consequences and giving negative attention to disruptive behavior, were each associated with more severe daily disruptive behavior. Here, adding within- and across-episode consistency to the model did not result in a significant improvement (Δ*F*(2,137) = 0.55, *p* = 0.581, Δ*R*^*2*^ = 0.01), indicating that the association between consistency and child disruptive behavior was explained by the individual negative responses. For model coefficients, see Table 3.

### Prediction of Externalizing Problems One Year Later

In Sample 1, we examined whether the consistency measures predicted externalizing behavior one year later, controlling for the severity of daily disruptive behavior at T1 and for parent-reported consistency as derived from a one-time questionnaire about general responses to tantrums. The first step was significant (*F*(4,73) = 5.01, *p* = 0.001, Δ*R*^*2*^ = 0.22): more severe daily disruptive behavior was predictive of more externalizing behavior one year later. Above and beyond this effect, less consistency as derived from parents’ reports of how frequently they generally displayed certain responses to their child’s tantrums (i.e. general consistency as computed from the baseline measure), was predictive of more externalizing problems. Importantly however, adding within- and across-episode consistency to the model did not result in a significant improvement (Δ*F*(2,71) = 0.001, *p* = 0.999, Δ*R*^*2*^ = 0.00). These results indicate that consistency in parental responses as derived from their reports of how often in the last month they displayed certain reactions, was longitudinally predictive of externalizing problems, whereas within- and across-episode consistency as derived from the daily diary measures were not. For model coefficients, see Table 4.

**Table 4:** Results of Regression Analysis Predicting Externalizing Behavior one year Later

variables	*b(SE)*	β	*p*
Step 1			
Age Child	-0.01(0.00)	-0.15	0.199
Sex Child	0.00(0.07)	0.01	0.955
Education Level	-0.01(0.03)	-0.04	0.676
Daily disruptive behavior severity T1	0.02(0.01)	0.31	0.005
General consistency	-1.90(0.68)	-0.31	0.006
Step 2			
Age Child	-0.01(0.00)	-0.13	0.260
Sex Child	-0.01(0.07)	-0.01	0.927
Education Level	-0.01(0.04)	-0.04	0.711
Daily disruptive behavior severity T1	0.02(0.01)	0.30	0.010
General consistency	-1.76(0.82)	-0.29	0.036
Within-episode consistency	0.05(0.09)	0.08	0.553
Across-episode consistency	-0.12(0.17)	-0.11	0.464

## Discussion

Aim of this study was to investigate how within- and across-episode parental consistency in responding to misbehavior are associated to externalizing problem behavior in children, using a daily diary approach. Within- and across-episode consistency were moderately strongly correlated with each other, but only across-episode consistency was associated with the severity of daily disruptive behavior. In Sample 1, this association was significant above and beyond the content of the parental reactions, whereas in Sample 2, the association was explained by the fact that parents who were more consistent across episodes were less likely to provide negative consequences or negative attention for the disruptive behavior. When we compared the longitudinal predictive value of the measures of consistency derived from the daily diaries to a measure of consistency derived from how often parents indicate they usually react, we found that the measures from the daily diary did not predict externalizing behavior problems one year later, whereas parental consistency as derived from the general questionnaire did.

### Within- and Across-episode Consistency

In both samples, we found that the two types of consistency were significantly associated with each other, but the associations were not so strong that they indicated that they reflected the same underlying construct. This indicates that some parents were relatively higher on across-episode consistency whereas others were relatively higher on within-episode consistency. These results support the idea that it is relevant to separate the two types of consistency. The correlations between the two types of consistency were quite similar across the two different samples, as were the associations between the two types of consistency as computed from the daily diary data and the ‘trait’ measure of consistency that was computed based on how parents indicated that they generally responded to disruptive behavior in the baseline measure. These associations provide some validation of these measures.

Interestingly, despite moderately strong correlations between within- and across episode consistency, when associations between the two types of consistency and the severity of daily disruptive behavior were examined, across-episode consistency was significantly associated with the severity of daily disruptive behavior in both samples, whereas within-episode consistency was not. That within-episode consistency was not associated with disruptive behavior severity is not in line with observational findings that lower within-episode consistency differentiated mother-child dyads with conduct-problems from those without (Gardner, 1989), and mothers of aggressive toddlers from those without (Del Vecchio & O’Leary, 2006). These findings may indicate that with regards to within-episode consistency, the rewarding nature of the final response – when a parent eventually gives in or does not follow through on their initial demand – is more important in explaining this effect of within-episode consistency rather than the mere variation of types of responses as was assessed by our measure. Parents who reward the child for misbehavior are likely to first provide negative attention for instance, scolding the child, and only give in after a sequence of different types of reactions (Gardner, 1989). Alternatively it may mean that across-episode consistency is actually more strongly associated with disruptive behavior. As previous studies examining these rewarding interaction sequences have not controlled for across-episode consistency, more research is necessary to examine whether this association also disappears when across-episode consistency is taken into account.

We add to previous findings that inconsistency in responses across episodes of misbehavior may be specifically associated with more severe disruptive behavior, regardless of the variation in types of responses within single episodes. In Sample 1, parents who were less consistent not only varied more within- or across-episode in how they responded to children’s tantrum, but also more frequently used each of the responses, both positive (e.g., positive consequences such as ‘giving in’) and negative (e.g., negative consequences such as ‘punishing’). Importantly, it was the variation between responses rather than the frequency of the individual responses that was associated with child disruptive behavior. This might indicate that parental consistency in responding is more important for lowering child disruptive behavior than how parents respond specifically. Alternatively, it might mean that when children show more disruptive behavior, parents are more likely to try out different ways of responding in an attempt to deal with it. Other studies have found that behavioral or emotional variation is associated with more maladjustment in young children as observed at a more micro time-scale, across real-time interaction. For instance, variability in affective displays has been related to more externalizing problems in mother-toddler dyads (Lunkenheimer et al., 2011), as has behavioral variability (Lunkenheimer et al., 2020).

Our findings support the idea that, in a non-clinical sample, predictable parental responses are most important in reducing disruptive behavior. Unpredictable behavior from parents has been shown to impact the stress response in infants, with a blunted cortisol response to a painful stressors for infants of mothers who’s behavior was less predictable (Noroña-Zhou et al., 2020), and variability in the affective quality of mother-child interactions and even in the timing of leisure activities, has been associated with an increased production of proinflammatory cytokines, an index of stress-reactivity, for youth (Manczak et al., 2018). More research is necessary to understand whether these processes play a role in explaining the association between across-episode consistency in parenting behavior and child disruptive behavior.

In Sample 2, a sample with older children who were at-risk for problem behavior, across-episode consistency was no longer associated with disruptive behavior above and beyond the individual reactions, whereas the frequency of providing negative consequences and negative attention were associated with more severe disruptive behavior. It seems that, whereas in Sample 1 it did not matter so much what parents did to reduce child disruptive behavior, as long as they did it consistently across episodes, in this sample negative responding was specifically associated with disruptive child behavior. Perhaps for families with older children with elevated levels of disruptive behavior as in Sample 2, parent and child have more strongly established patterns of negative responding to each other (Granic & Patterson, 2006). In support of this idea, in studies of school aged children, affective variability has been associated with less rather than more behavioral problems (Granic et al., 2007; Hollenstein et al., 2004). Settling into a rigid, negative interaction style is a process that takes place in the interaction between parent and child over several years. Heightened variability in other areas may still have negative effects in older children and adolescents. For instance, higher variability in experienced stressors has been associated with worse emotional adjustment in adolescents (Zheng et al., 2022), and higher variability in daily activities is associated with lower psychological well-being in young adults (Lee et al., 2018).

### Daily Diary Measures

In this study, daily disruptive behavior was associated with externalizing problems one year later, and within- and across-episode consistency were associated with our measure of general consistency. Thus, it appears that the daily diary measures were tapping some of the micro-level processes giving rise to increases in problems at a developmental timescale (Granic & Patterson, 2006). However, it also appears that these associations between parenting and child behavior did not cross-over from one level to the other, as the general parental consistency measure was predictive of externalizing problems one year later, whereas the daily measures of consistency (within- and across-episode consistency) were not. Additionally, general consistency was in turn not associated with daily disruptive behavior severity, whereas the daily measure of across-episode consistency was. It thus seems that the parenting and child behavior measures that were measured on a more similar timescale were more likely to be associated with each other. A previous study had similar findings in this regard, with daily measures of parenting variability associated with global parenting measures, but only global measures associated with a measure of the child’s ADHD symptoms (Li & Lansford, 2018). Although there, ADHD symptoms became significantly associated with variability in parental warmth after controlling for parental ADHD symptoms and several types of stress, and daily symptom expression was not assessed. More research is necessary to understand how inconsistency in daily parent-child interactions may eventually increase externalizing problems over months and years.

Although daily diary measures are especially helpful in differentiating within- from across-episode consistency, the measure of how often parents indicated to react to their children’s disruptive behavior a certain way over the past month – the general consistency measure – was more predictive longitudinally of externalizing behavior than the daily diary measures. Although this measure again confounds within- and across-episode consistency, it may still be a better measure of consistency than some of the other measures of general consistency. As we asked directly about very specific reactions, our measure is likely a more valid measure of actual consistency in responding (Morsbach & Prinz, 2006). At the same time, we would like to note that the validity of this measure deserves further scrutiny.

### Strengths and Limitations

This study has several strengths. First, we included two samples of daily diary data that allowed us to differentiate within- from across-episode consistency and examine how our results would replicate across samples. Second, the analyses were registered on the OSF before conducting them. In addition to these strengths, some limitations are also worth mentioning. First, we did not differentiate different types of disruptive behavior episodes. Perhaps some episodes were more similar to each other than others, with a child yelling after not getting what it wanted in separate instances more similar than hitting a sibling in frustration about losing a game. Parents might react differently to different types of misbehavior, but consistently so within the types of misbehavior. Relatedly, in the functionally based categorization of behaviors in this study, certain responses were collapsed into categories as they were highly similar in their function – with these categorizations preregistered. Categories of positive attention, and ‘getting what you want’ were differentiated as they are likely different enough to be inconsistent, as are receiving negative attention or being punished for instance. An even higher level of abstraction could also be chosen, where anything ‘positive’ is contrasted with anything ‘negative’. At present, it is not known how different responses must be to contribute to inconsistency. Relatedly, the categorization of the parental reactions was based on a social learning theory perspective. However, inconsistencies in other aspects of the response might also be relevant. For instance, for several of the reactions that parents could choose from, it would be possible to be quite calm or quite frustrated while doing so, and these differences in affective quality and intensity might also contribute to inconsistency. More research is necessary to investigate whether inconsistency computed from other aspects of parental responses shows similar associations as the inconsistency measures derived from the categorization we made here. Second, both samples consisted of families with mostly highly-educated parents, raising questions about how generalizable these findings are to populations with different educational backgrounds. Additionally, whereas for Sample 2 it was clear that it was representative of ethnicities in the Netherlands, for Sample 1 information about ethnic diversity of the sample was not collected, making it impossible to draw any conclusions about this. Third, although similar measures were available for both samples included in this study, these studies were not designed to be the same, and as a result varied in multiple design aspects, making it impossible to draw any conclusions about why the results may have differed between them. Fourth, there are other aspects of consistency that we have not included in this study. For instance, consistency between different caregivers’ reactions might also play a role adjustment problems (Dwairy, 2010).

### Conclusion

Results of this study show that it is meaningful to separate parental consistency within- from consistency across-episodes of misbehavior as they are correlated, but not strongly so. Furthermore, the aspects of consistency may be differentially important for the severity of child disruptive behavior as it is displayed in daily life, and there is some indication that across-episode consistency might be more important than actual responses, at least in a general population sample of toddlers. However, the actual responses were more important in our sample of early elementary school aged children from an at-risk population. Findings thus suggest that different risk factors (across-episode consistency or negative responding specifically) for disruptive behavior might apply to different subpopulations.

## Data Availability

Data are available from the first author upon request.

## Appendix I: Questions as included in the daily diaries

### Parental Reactions

#### Sample 1

For each tantrum during that day in daily diary, question: *‘How did you react to tantrum x?’ (x = 1–7 max)*

#### Sample 2

For the most difficult to manage disruptive behavior reported that day, question: *‘How did you react to this disruptive behavior?’*

For answer options, see Table 5. Answer categories for each option in both samples were: check box, coded unchecked = 0, checked = 1. Multiple checks allowed.

**Table 5:** Answer categories for each parental reaction for the two studies

**Study 1**	**Study 2**
**Negative consequence**	
I sent my child to their room/corner/time-out	I sent my child to their room for at least an hour
I punished my child	I gave my child a short time-out, away from others
	I took something nice away from my child (e.g., toys or screen time)
	I gave my child extra chores (e.g., set the table)
**Positive consequence**	
I negotiated with my child	I gave my child his/her way
I gave in to my child	I gave in to my child
**Withholding attention**	
I didn’t, I let my child cool off	I did nothing
I ignored my child	I talked about it with my child afterwards
**Negative attention**	
I became angry with my child	I yelled/swore
I grabbed my child	I said things I didn’t mean
I spoke sternly to my child	I threatened with punishment, but did not punish
**Positive attention**	
I comforted my child	I begged my child to stop
I distracted my child	I used humor to distract my child

### Child Externalizing Behavior

#### Sample 1

For each tantrum that occurred that day, question: ‘*Which of the following behaviors did your child show during tantrum x?’ (x*=*1-7)*

For answer options, see Table 6. Answer categories for each option in both samples were: check box, coded unchecked = 0, checked = 1. Multiple checks allowed.

**Table 6:** Answer categories for the aggressive tantrum behaviors

Hitting	
Kicking	
Biting	
Throwing an object	
Pushing/pulling	
Spitting	
Grabbing	
Banging head	
Holding breath	
freezing	

#### Sample 2

Question: ‘*How disruptive was your child’s behavior today?’.*

Answer categories were: slider 1 − 10.
